# What if something happens tonight? A qualitative study of primary care physicians’ perspectives on an alternative to hospital admittance

**DOI:** 10.1186/s12913-021-06444-x

**Published:** 2021-05-11

**Authors:** Vivian Nystrøm, Hilde Lurås, Patrik Midlöv, Ann-Chatrin Linqvist Leonardsen

**Affiliations:** 1 Department of Health and Welfare, Østfold University College, (PB) 700, 1757 Halden, Norway; 2 Health Services Research Unit, Akershus University Hospital, (PB) 1000, 1478 Lørenskog, Norway; 3 Institute of Clinical Medicine, Campus Ahus, University of Oslo, Lørenskog, Norway; 4 Center for Primary Health Care Research, Department of Clinical Sciences Malmö, Lund University, (PB) 50332, 202 13 Malmö, Sweden; 5 Østfold Hospital Trust, Halden, Norway

**Keywords:** General practitioner, Primary care physicians, Health services research, Interview, Primary healthcare, Quality improvement

## Abstract

**Background:**

Due to demographic changes, hospital emergency departments in many countries are overcrowded. Internationally, several primary healthcare models have been introduced as alternatives to hospitalisation. In Norway, municipal acute wards (MAWs) have been implemented as primary care wards that provide observation and medical treatment for 24 h. The intention is to replace hospitalisation for patients who require acute admission but not specialist healthcare services. The aim of this study was to explore primary care physicians’ (PCPs’) perspectives on admission to a MAW as an alternative to hospitalisation.

**Methods:**

The study had a qualitative design, including interviews with 21 PCPs in a county in southeastern Norway. Data were analysed with a thematic approach.

**Results:**

The PCPs described uncertainty when referring patients to the MAW because of the fewer diagnostic opportunities there than in the hospital. Admission of patients to the MAW was assumed to be unsafe for both PCPs, MAW nurses and physicians. The PCPs assumed that medical competence was lower at the MAW than in the hospital, which led to scepticism about whether their tentative diagnoses would be reconsidered if needed and whether a deterioration of the patients’ condition would be detected. When referring patients to a MAW, the PCPs experienced disagreements with MAW personnel about the suitability of the patient. The PCPs emphasised the importance of patients’ and relatives’ participation in decisions about the level of treatment. Nevertheless, such participation was not always possible, especially when patients’ wishes conflicted with what PCPs considered professionally sound.

**Conclusions:**

The PCPs reported concerns regarding the use of MAWs as an alternative to hospitalisation. These concerns were related to fewer diagnostic opportunities, lower medical expertise throughout the day, uncertainty about the selection of patients and challenges with user participation. Consequently, these concerns had an impact on how the PCPs utilised MAW services.

**Supplementary Information:**

The online version contains supplementary material available at 10.1186/s12913-021-06444-x.

## Background

The increased proportion of older adults and people with chronic diseases in the general population has resulted in an increased demand for health services worldwide [1–3]. Hospital emergency departments in several high-income countries are overcrowded due to the large proportion of non-urgent patients [4–6]. Many countries are launching primary healthcare models as alternatives to hospitalisation, as well as aiming to increase coordination across healthcare levels to improve patient care, reduce costs and improve public health [7–10]. Community hospitals, observation wards and hospitals-at-home are examples of health service models at the interface between primary and secondary care that provide acute and/or non-acute services and offer a variety of treatment and diagnostic services [11, 12].

In Norway, health services are primarily divided into two levels: primary care services, including general practice, out-of-hours emergency services offered in casualty departments where patients may be assessed by primary care physicians, home-based care, nursing homes and municipal acute wards (MAWs), and specialist services, including hospitals, outpatient specialist care and contract specialists [13]. The MAW model has been in use since 2012 as an alternative to hospitalisation for adult patients who need acute treatment and care but not specialist health services [13, 14]. MAWs are located in the local community, near where people live. The inclusion criteria for admission to a MAW are as follows: patients who would otherwise be admitted to the hospital; maximum expected length of stay of 72 h; acute deterioration of a known, chronic condition; and other clarified conditions where the risk of acute deterioration is low. Eligible patients must be aged above 18 years [15]. In 2019, there were 216 MAWs in Norway, with a total of 735 beds [16]. MAW beds are located either inside nursing homes, in casualty departments or in separate units at local medical centres. MAWs range from small units with 3 or fewer beds to the largest units with 15 beds or more [16]. Studies have indicated that the MAW model is a good alternative to hospitalisation and that patients prefer to be admitted to a MAW rather than the hospital [17–20]. Moreover, a study indicated that implementation of MAWs led to a 1.9% reduction in hospitalisations for patients aged over 80 years [14].

In many countries, primary care physicians (PCPs) are assumed to be gatekeepers responsible for assessing patients within the catchment area of their practice and for referring them to specialist health care services [21]. Since 2001, when a list patient system was implemented, Norwegian inhabitants have the right to be listed with a specific PCP [22]. Outside of office hours, patients can also access PCPs in a casualty department, where the PCP on duty does not necessarily know the patient or have access to his or her medical records [23].

Previous studies have indicated that PCPs’ attitudes towards the development of health systems and the treatment of fragile patients may be barriers to the implementation of alternatives to hospitalisation [24–26]. Hence, for the implementation of new health service models, it is important to gain knowledge about the aspects that PCPs find important when referring patients to different healthcare services. Studies focusing on PCPs’ perspectives on alternatives to hospitalisation are lacking. Consequently, the aim of this study was to explore PCPs’ perspectives on the MAW model as an alternative to hospitalisation.

## Materials and methods

This qualitative study utilised semi-structured interviews with PCPs. The study adheres to the Consolidated Criteria for Reporting Qualitative Research (COREQ) guidelines [27].

### Setting and participants

The study was performed within one hospital catchment area in a county in southeastern Norway with approximately 320,000 inhabitants. In total, there are 288 PCPs in the area working in private practice and/or in casualty departments. The county includes five MAWs with four to eleven beds. Three MAWs are located within a casualty department, and two MAWs are located 5 to 15 min from a casualty department. The staff consists of registered nurses, specialist nurses and physicians; nurses are present all day and night, and physicians are present during the daytime on weekdays. MAW personnel can take blood samples to be analysed in the hospital laboratory, and it takes about 2 days to get the results back. In four of the MAWs, X-ray services are available in the daytime.

It is possible for PCPs and MAW personnel to send patients to the hospital for extended diagnostics before admittance to a MAW, which is called a ‘diagnostic loop’. Patients are sent by taxi or ambulance to the hospital’s emergency department to provide blood samples, undergo X­rays or ultrasound scanning, or be assessed by hospital specialists before being transferred to a MAW. The MAWs operate according to a timeframe of a maximum of 6 h, within which the hospital must confirm the patient’s transfer back to the MAW.

### Data collection

Strategic and snowball selection methods were used to recruit PCPs from both rural and central areas of the county [28]. The PCPs received information about the study and an invitation to participate by email, which was forwarded from the head PCP in each of the municipalities. Based upon suggestions from the study nurses, potential participants were contacted by the first author either in the clinic, by phone or by email. Five PCPs refused to participate. After accepting an invitation, no PCPs withdrew their consent to participate. We aimed to achieve a maximum variation sample [28], including by geographical location, gender, age, years of experience as a physician and years of experience working in a hospital (if applicable) (see Table 2). Recruitment continued until the first and last author agreed that data saturation had been achieved, meaning that no new themes were identified in subsequent interviews [28].

An interview guide (Additional file 1) was developed in accordance with the literature on healthcare quality at different healthcare levels, patient satisfaction, healthcare status, outcome measures and patient-centred care [18, 19, 29–31] and through iterative discussions between the authors until consensus was reached. The guide was pilot-tested for content and face validity through interviews with two experienced PCPs (both male), and small changes were made. For example, we elaborated on question 4, which initially read, “Could you please describe the admission process”, adding “… who do you contact, what kind of documentation is needed?”

Semi-structured interviews were conducted by the first author in the PCPs’ offices from April to August 2020. The interviews lasted from 22 to 56 min, with an average duration of 38 min. All interviews were digitally recorded and transcribed verbatim by the first author within 2 days.

The research group included a critical care nurse/PhD candidate, a nurse anaesthetist/PhD, a physiotherapist/professor, and a PCP/professor, with one male and three females.

### Analysis

A thematic analysis with a reflective approach in line with recommendations from Braun and Clarke [28, 32, 33] was performed. The first author’s own thoughts and ideas were written down before and immediately after each interview. The purpose was to explore any dynamics and behaviour between the interviewer and the participant that could potentially impact the analysis. The reflection notes were read together with the transcripts and incorporated into the analysis through several discussions between the authors.

The interview transcripts were inductively analysed using a six-phase process. More specifically, the first phase included familiarisation with data through listening to the recordings (first author) and reading and re-reading the transcripts (first and last author). In phase two, the first and last authors inductively coded the transcripts individually, and then the codes and initial themes were discussed until agreement was reached. In phase three, all authors were involved in searching for themes. The second and third authors sent overviews and reports via email, and the initial themes were discussed in virtual meetings. In this phase, Word and Excel were used as tools to structure the data material, and no further computer-assisted qualitative analysis software was utilised. During phase four of the analysis, the themes were reviewed. Some themes were merged, and some were divided. The first and last authors went back and forth between the transcribed material and the subthemes and themes, as well as the impressions from the first authors’ reflexivity notes, to identify the content and totality of the data. All authors then discussed the themes in relation to the whole data set, also keeping the first authors’ reflections in mind, as a process to increase the robustness of the analysis. In phase five, we identified and named the final two themes and three subthemes that were related to the aim of the study. In the sixth phase, the paper was written (see Table 1).

**Table 1:** Thematic analysis according to Braun and Clarke, 2006

Transcript	Codes	Subtheme	Theme
PCP 2: They (the physician and nurses at the MAW) often say that this is not a MAW patient. I have come across that quite often. Interviewer: Do you say any more about that then? PCP 2: Because they (the physician and nurses at MAW) believe this one ought to be in hospital. As a rule, you try to get a diagnostic loop instead. So then you meet them kind of halfway. Sometimes I can understand that too. They (the patients) have so much different stuff … and where does one begin … and then they might need a specialist for that. Interviewer: Too many of those patients have comorbidities. PCP 2: Yes. But sometimes I feel like this might be maybe... They (the patients) have a number of conditions. This is what they need help with right now. Not everything else.	Not a MAW patient Quite often The physician and nurses at the MAW believe the patient should be admitted to hospital Diagnostic loop Meet halfway Patients have a lot of different stuff (comorbidities) Where to start? Need a specialist Patients have a number of conditions This is what they need help with now Not everything else	Safety for all (here: for the admitting PCP, as well as for the physician and nurses at the MAW)	What if something happens tonight?

*Abbreviations*: *PCP* Primary care physician, participant number, *I* Interviewer

## Results

A total of 21 PCPs agreed to participate in the study: 12 males and nine females. The PCPs’ mean age was 39 years, their mean work experience as a physician was 11 years, and their mean work experience at a hospital was 2 years (Table 2).

**Table 2:** Information on the study participants’ gender, age, years of work experience as a physician, and years of experience from working in hospital at the time of data collection

Number	Gender	Age	Qualified physician	Work experience in a hospital
PCP 1	M	29	2	1
PCP 2	F	34	4	1
PCP 3	M	33	8	1
PCP 4	F	39	14	1.5
PCP 5	F	41	14	2.5
PCP 6	M	35	9	2.5
PCP 7	F	32	6	1
PCP 8	F	41	16	1
PCP 9	M	40	15	4
PCP 10	M	42	13	2.5
PCP 11	M	42	15	2
PCP 12	M	60	20	1
PCP 13	M	41	9	3.5
PCP 14	M	42	15	6
PCP 15	F	41	15	2
PCP 16	M	31	6	1
PCP 17	F	30	5	2.5
PCP 18	F	52	17	3
PCP 19	M	32	6	2.5
PCP 20	F	32	3	1.5
PCP 21	M	44	20	2

*Abbreviations*: *PCP* Primary care physician, *F* Female, *M* Male. Age = reported in years. Qualified physician = reported as years of work experience since graduation. Work in hospital = years of work experience from hospital

The findings indicate that the PCPs had different views on which level of healthcare service a MAW represented. MAWs were described as a “mini-hospital”, a “peripheral hospital unit”, a “specialised nursing home” and a “primary healthcare service level”. Despite varying descriptions of MAWs, most of the PCPs had a clear idea of “the typical MAW patient”, frequently described as an elderly patient with an acute need for treatment with a higher level of health services. Two main themes were identified through the analysis. The first theme was “What if something happens tonight?” The subthemes were a) safety for all, b) competence and responsibility, and c) collaboration across health service levels. The second them was “User participation”.

### What if something happens tonight?

The PCPs’ main concern was related to what could happen if a patient who would otherwise have been hospitalised deteriorated after admittance to a MAW. All PCPs emphasised that it was important that healthcare services provided safety for themselves, for the personnel at the MAW and for the patient. The PCPs’ concern was also related to issues of staff competence at the MAW, as well as the distribution of responsibility and collaboration among health service levels.

#### Safety for all

There were great variations in whether the PCPs felt that admission to a MAW was a medically safe alternative to hospitalisation. Most PCPs reported uncertainty in making a tentative or preliminary diagnosis based on their time-limited judgement, sparse medical records, few laboratory tests and the patient’s own narrative. Many of the PCPs highlighted that they felt safer referring patients to a MAW when utilising the diagnostic loop at the hospital first, enabling, for example, extended blood testing or X-rays. In addition, many PCPs felt that it was safer for a physician at the hospital to perform a second assessment of the tentative diagnosis. An experienced PCP stated, “I also feel more secure about a patient who I admit to the MAW when he has been through a diagnostic loop … I feel that it’s a very good service … the patient will have even more clarity, and the MAW will receive a treatment plan from the hospital” (PCP four). This excerpt indicated that the PCP found admission to a MAW to be riskier for the patient, as well as for herself, than admission directly to the hospital.

The PCPs felt responsible legally, medically and ethically for making the right decision. It seems that it was easier for the PCPs to place trust in the hospital personnel’s diagnostic decisions and further treatment plans than to diagnose the patient herself, which would carry the possibility of making a mistake. However, many of the PCPs also emphasised that ‘the diagnostic loop’ involved a further need for transport to the hospital and that patients would then be exposed to long waiting times in the emergency department at the hospital. They also stated that many elderly patients are cognitively disoriented and that the hospital atmosphere would negatively affect their condition, which might present an increased risk for the patient.

All of the PCPs reported that a clear diagnosis with a clear treatment plan was a criterion for MAW personnel to feel safe enough to accept the patient. The PCPs found providing a clear diagnosis challenging, arguing that a medical diagnosis is associated with a probability of misinterpretation. Hence, the PCPs often sent a patient through a diagnostic loop, even if they did not find it necessary, just to make the MAW personnel feel safe.

Many PCPs also stated that patients who had previously spent time at an MAW wanted to be admitted there rather than to the hospital. In particular, this desire was often held by patients with deterioration of a chronic condition or by frail elderly patients with multimorbidity. The PCPs noted that some of their patients had reported back to them about negative experiences at the hospital, such as waiting for hours in an overcrowded emergency department or being placed in a corridor. The PCPs suggested that being admitted to a MAW provided a sense of security for these patients. PCP 5 spoke in a low but clear voice as she conveyed the following message: “I think it’s the fact that it’s local, so that the family is able to visit, and, among other things, that the rooms are pleasant and spacious …. there are lots of those practical things … but they also feel really safe and cared for … this is very important” (PCP five).

Hence, the PCPs found it important that healthcare services feel safe for both the referring and treating physicians, as well as for the patient.

#### Competence and responsibility

Most of the PCPs were concerned about competence or lack thereof among MAW personnel. Due to the relatively high morbidity among patients eligible for admittance to a MAW, most of the PCPs emphasised a preference for a wide range of medical expertise among personnel working at a MAW. Many of the PCPs indicated that due to the need for advanced medical treatment in primary healthcare, MAWs should ideally be staffed with hospital physicians. Others stated that a MAW should be staffed with PCPs or geriatricians. They also reported that it might be demanding for inexperienced physicians to have the sole medical responsibility at a MAW, although this was often the case. Most of the PCPs had earlier work experience from a hospital and contrasted the lonely physician role at a MAW with team-based collaboration in a hospital. PCP 21 thought about this issue for a while and then said, “I think perhaps that a background in internal medicine … But then general medicine can be a very good background for working there too. Although it should be clarified, there can still, for example, be things that aren’t clear-cut, and then it is important to have that breadth” (PCP 21). The PCPs assumed that it would be quite demanding for a physician to be in charge of a MAW. Hence, they believed there should be a minimum competence required to work in a MAW.

Most of the PCPs reported that they found it to be a challenge that the MAWs were staffed by nurses all day and night, with physicians present only during the daytime. This staffing arrangement resulted in a lack of medical expertise outside of ordinary working hours and thereby limited opportunities to re­evaluate the patient’s medical diagnosis. The PCPs with experience working in a hospital also considered physicians’ medical decision-making role in hospitals. Therefore, most PCPs felt that MAW patients were their responsibility in the evening and at night. PCP 20, for example, thought that this was a very heavy burden: “Imagine if a patient’s condition suddenly worsens and that is not discovered! I think that is actually a major barrier” (PCP 20). This excerpt indicated the PCPs’ lack of trust in the competence of nurses at MAWs. In contrast, the nursing services were described as generally good by most of the PCPs, especially regarding their ability to provide structured observations and basic care. Nevertheless, some of the PCPs experienced a lack of preparedness in acute situations; as PCP 19 noted, “You do not see the really serious cases at the MAW; they are in hospital. I have been called out (to see a patient), and the opposite has been the case. They (the nurses) concluded that it is very serious. Then, you find yourself standing there, and the patients aren’t so critical after all ….” (PCP 19). This PCP believed that the nurses’ competence in handling acute cases was better at the hospital than at the MAWs.

#### Collaboration across health service levels

The PCPs perceived that the MAWs operated at the interface between the hospital and primary healthcare services but were still at a lower health service level than the hospital. They also reported several barriers to collaboration between the two health service levels. The collaboration was described in different terms, such as that the “systems do not talk to each other”, that there are “bottlenecks in the system” and that there is “a tiresome bureaucracy”.

Collaboration between PCPs and hospital physicians was assumed to be essential to clarify the patient’s condition and to establish a mutual understanding of one another’s situations. Nevertheless, the PCPs described a hierarchical relationship between hospital physicians and PCPs. Some of the PCPs described hospital physicians as specialists and as therefore being able to provide decisional support. However, due to national legislation, PCPs have a legal right to refer patients to the hospital even if hospital physicians disagree. PCP 11 stated clearly, “Sometimes, there can be a great deal of discussion regarding patients. But I’ve been working with this for so long now, so on the whole, I’ve made my decision if I call … and if there will be a discussion (with the hospital personnel), I just say we can agree to disagree about this … but the patient comes to you anyway. It is my privilege to be able to make referrals, and they are obliged to make an assessment” (PCP 11). Hence, this excerpt suggested an imbalance in the relationship between hospital physicians and PCPs created by two different traditions: primary care and specialist health care.

In contrast, the collaboration between MAW physicians and PCPs was described as two-way communication about risk assessment, tentative diagnoses and treatment plans. However, some of the PCPs also reported disagreements based on different interpretations of MAW admission criteria and the clarify of the patient’s condition. PCP eight described this issue as follows: “So that’s generally what we find a bit difficult when there are admissions to the MAW … there are some restrictions and … a bit difficult … they admit patients … but their requirements are more demanding regarding the kind of patients they will admit” (PCP eight). This quotation indicated that the collaboration between PCPs and MAW physicians also involve some challenges.

In the evening and at night, the PCPs had to communicate with the nurses at the MAW when admitting a patient. Many of the PCPs described this communication as somewhat more complicated than communicating with a physician colleague. In these situations, the PCPs encountered even more discussions and disagreements, which they interpreted as nurses feeling uncertain about whether they should accept the patient. Some of the PCPs attributed this disagreement as nurses and physicians not speaking the same language, requiring both the patient’s medical condition and treatment to be explained in a simplified way when a PCP communicated with a nurse. This suggests that physicians and nurses had different needs. The nurses wanted the PCPs to develop a treatment plan, and the PCPs wanted a colleague to discuss both treatment and diagnosis.

### User participation

When deciding the level of treatment, the PCPs found it important to determine whether the patient was able to provide consent and expressed frustration with patients and relatives who had unrealistic expectations regarding treatment and admittance. This was especially challenging when the PCPs encountered conflicts with their assumptions of what constituted professionally sound practices.

The PCPs stated that it was important to involve the patient in his or her own treatment. Mapping the patient’s expectations and desires was assumed to be key to supporting the patient’s true participation. Some patients clearly requested to be admitted to the MAW rather than to the hospital. The PCPs then found it important to identify the reason why the patient required admission to a MAW or to the hospital. The PCPs highlighted good communication as being key to making the best choice for the patient and noted that good communication required that the patient be able to provide consent. PCP 17 thought about this topic for a while and then said, “One tries as best as one can to comply with the patients’ wishes as well … that obviously means a lot... I quite often ask what they are imagining … what they are thinking … whether they are scared … or what … are they worried about staying at home …” (PCP 17). For this PCP, involving the patient in her assessment was assumed to be essential.

Many of the PCPs also stated that relatives often had a central role in communicating the patient’s wishes and needs. The PCPs assumed that relatives may be able to provide more complete and comprehensive descriptions of the patient’s habitual condition than patients themselves. PCP five explained this assumption in this way: “Call relatives …. also get a realistic impression of their functional status. I actually think that many of those who come here are in a poor condition. But maybe they are like that all the time, so is there such a great difference?” (PCP five). Nevertheless, due to both the time of the day and the limited amount of time to make a decision, including relatives was not part of the PCPs’ usual practice.

In many cases, the PCPs reported that many patients would not have survived at home without relatives compensating for the patient’s frailty due to old age, multimorbidity and a low level of functioning. The PCPs explained that it was not always that the patient wanted to be admitted to a MAW or hospital but that relatives did not have the capacity to take care of the patient at home anymore. Hence, the PCPs described that they had to take both patients’ and their relatives’ wishes into consideration when deciding the level of treatment.

## Discussion

This study contributes knowledge about PCPs’ perspectives on the MAW model as an alternative to hospitalisation. Several factors affected the participating PCPs’ judgement of whether the use of MAWs is a safe alternative to hospitalisation, both from their own perspectives and from those of MAW personnel, patients and patients’ relatives. Moreover, disagreements regarding admission criteria, concerns related to the competence of MAW personnel, the unclear distribution of medical responsibility and collaborative challenges between primary and hospital health service levels all impacted the PCPs’ decisions on where a patient should be treated. Involving patients and relatives in the decision about the treatment level was also assumed to be important but challenging.

The PCPs were worried about what would happen if a patient’s condition deteriorated in the evening or night when there was no physician on duty at the MAW or if the PCP had misjudged the situation. A recent study of 2744 admissions to a MAW showed that 23.6% of admitted patients were transferred to the hospital as a result of deterioration or the identification of a clinical condition that required hospital care [34], which underlines the PCPs’ worry. To compensate for the PCPs’ insecurity and to avoid later hospitalisation, the diagnostic loop was considered a good solution. In addition, ‘the loop’ was requested by physicians and nurses at the MAWs. In contrast, a recent study found that PCPs felt that the diagnostic loop was an inappropriate use of time and resources [31]. Managing diagnostic uncertainty has been reported as a recurring challenge for physicians [35]. Several studies have shown that more experienced PCPs tolerate more risks and diagnostic uncertainty than other physician groups, who rely more on examinations to make a diagnosis [9, 36, 37]. This was not in line with findings in a recent study.

The PCPs reported that many patients wanted to be admitted to a MAW, especially older adults who had been to a MAW before. Such positive patient experiences of MAWs have also been highlighted in other studies [18–20]. However, a Cochrane review also showed that patients with acute illness were satisfied with receiving treatment in primary care after a short hospitalisation [38]. Hence, the issue of whether patients who are acutely admitted to either a MAW or a hospital are able to objectively evaluate the services separately should be examined. It may also be argued that a patient’s condition when treated in an MAW is less acute and therefore impacts his or her impression of the stay.

In cases of deterioration or misjudged conditions, the PCPs were not convinced that admission to a MAW was a safe alternative to hospitalisation. They reported that they would prefer that MAWs be staffed by hospital physicians or medical specialists, and they had the impression that MAW physicians were quite inexperienced. It may be argued that MAW physicians could also be described as PCPs due to their role in primary healthcare. Hence, this may indicate a lack of insight from the PCPs into the actual competence of MAW physicians. A comparison of PCPs and hospital physicians in hospitals-at-home in Denmark showed that the PCPs managed to avoid the hospitalisation of elderly patients with acute medical diseases to a greater extent than hospital physicians [37]. This finding indicates a gap between PCPs’ assessments of their own, MAW physicians’ and hospital physicians’ competence.

Moreover, the PCPs in this study felt insecure because nurses staffed the MAWs for 24 h, while physicians were present only during the daytime. Vatnøy and colleagues found that reliance on nurses in acute inpatient settings requires that they have expertise to identify problems and take action [39]. The nurses in the MAWs were all registered nurses with a bachelor’s degree, and many of them had a specialisation, e.g., in critical care, geriatrics or diabetes. In addition, many of the nurses had considerable experience working in hospitals before they started working at the MAW. Hence, the PCPs seemed to lack insight into the nurses’ educational backgrounds and experience. Johannessen and Steihaug found that a lack of medical expertise was perceived as stressful and unsafe for nurses working in MAWs [31]. A Cochrane review indicated that nurse-led health services provide similar or better health outcomes, such as reduced mortality or reduced symptom burden for patients, than physician-led health services [40]. Regardless, neither the PCPs in the current study nor the personnel at the MAWs deemed nurses’ competence to be sufficient to provide safe and quality health services. This finding may indicate that it is time to reconsider how MAWs or similar primary care alternatives to hospitalisation should be staffed in the future. In addition, this finding indicates a need to better inform physicians about nurses’ competence, and vice versa.

The finding on PCPs’ feelings of insecurity when admitting a patient to a MAW is supported by, e.g., Leonardsen et al. [29]. Nevertheless, another study indicated that there were no differences in mortality and morbidity after patient admission to a MAW than after admission to a hospital [17]. Therefore, research on these primary healthcare alternatives to hospitalisation is essential to ensure that the different health care levels are used as intended. The findings in our study show that hospital physicians were considered medical specialists, while MAW physicians were considered collaborative partners. This finding was also reported in another study of general practitioners’ perspectives on patient handovers in primary healthcare [41]. The Vanguard study in England indicated that collaboration is difficult because there are barriers between the different health care levels [42], which is supported by our findings. A lack of communication and collaboration between healthcare providers is one of the main factors for stress among PCPs in high-income countries [43]; hence, this situation demands action. A focus on better collaboration between primary healthcare services and hospitals is essential to ensure the exchange of knowledge and experiences related to various diseases and how they are handled [44, 45].

The PCPs in our study reported that patients’ and relatives’ participation in deciding the level of healthcare services was important but challenging. This finding is in line with findings of recent studies [30, 46]. Patients’ involvement in decisions about their own health is associated with improved healthcare outcomes and patient satisfaction [47–49]. Such involvement requires that physicians ensure that the patient is cognitively oriented and sufficiently informed [50], which was also noted by the PCPs in our study. Since many older patients are cognitively impaired, it can be very difficult for them to be involved in accordance with a patient-centred care model [30, 51]. Conflict of the wishes of patients and their relatives conflict with what PCPs consider to be the best medical decision can result in PCPs taking a more biomedical view rather than a patient-centred care approach when deciding the medical treatment [52]. PCPs have an obligation to not only ensure the patient’s medical safety but also involve the patient in his or her own medical treatment [49, 53]. Healthcare professionals report that patients have unrealistic expectations of what is feasible [54], which is supported by our findings. Thus, there is a potential ethical conflict between shared decision-making, on the one hand, and patient safety, on the other [53].

The implementation of MAWs in Norway was based on a political initiative, and important stakeholders, such as PCPs, were accorded minimal involvement in the development of the model. In a constantly developing healthcare system, organisational change is more likely to succeed if healthcare professionals have an impact on the changes [55]. The participating PCPs’ uncertainty and concerns probably resulted in patients being referred to the hospital instead of the MAWs. Therefore, in the further development of the MAW model, it is important to consider these aspects to ensure quality services and safety for all stakeholders.

### Strengths and limitations

The qualitative research design entails a lack of opportunity for generalisation. Although the MAW model may appear to be a Norwegian concern, similar models have been developed in many other Western countries. This supports the transferability of our findings [11, 12]. Transferability is also supported by the use of the maximum variation approach, ensuring the participation of PCPs from both rural and central geographical locations variation in the PCPs’ age, gender, work experience as a physician, and experience working in a hospital.

A strength was that the interview guide was pilot tested by two experienced PCPs. A thorough transcription of the interviews was conducted: the PCPs’ stories were written down verbatim, including both verbal and non-verbal utterances, ensuring the internal validity and consistency of the findings. The first author’s reflexivity notes were consulted throughout the analysis process, and all authors were involved in the discussion of codes, subthemes and themes, thereby achieving confirmation.

Credibility refers to confidence in the “truth” of the findings [28], which was achieved through the thorough description of the data collection and analysis and transparency. The research group had a broad composition, with members of different genders and roles. The presence of different genders reduces gender bias, and our different roles contributed to a broader interpretation of the analyses of the PCPs’ perceptions of the MAWs, also increasing the credibility of our findings.

## Conclusion

The PCPs in our study reported several concerns related to the security of patients, MAW personnel and themselves in the consideration of admission to a MAW as an alternative to hospitalisation. Their concerns had an impact on how MAW services and hospital services were utilised. Moreover, user participation was assumed to be challenging due to conflicts between patients’ and relatives’ desires and what the PCPs found professionally justifiable.

Our findings indicate that PCPs do not have an overview of the competence of MAW personnel and resources of MAWs. For new health service models to be implemented and utilised as intended, our findings indicate that including key personnel perspectives is essential. Hence, it is important to increase the flow of information about alternatives to hospitalisation. It is also important to control whether this information is actually received.

## Supplementary Information

**Additional file 1.**

## Data Availability

Datasets generated and/or analysed during the current study are not publicly available due to local ownership of data, but aggregated data are available from the corresponding author on reasonable request.
