# Supplementary material for: What if something happens tonight? A qualitative study of primary care physicians’ perspectives on an alternative to hospital admittance
Source: BMC Health Serv Res. 2021 May 11;21:447. doi: 10.1186/s12913-021-06444-x (PMC8112060; doi:10.1186/s12913-021-06444-x)
Supplement: Supplementary file 1 — Additional file 1. [file 12913_2021_6444_MOESM1_ESM.docx]

**Interview guide**

Admission of patients

1. How would you describe a typical MAW patient?
2. Which are your criteria for admitting a patient to a MAW?
3. Can you please describe the situation the last time you admitted a patient to the MAW?
4. Can you please describe the admission process – who do you contact, what kind of documentation is needed?
5. Which eventual challenges do you encounter when admitting a patient to the MAW?
6. Which eventual challenges do you encounter when admitting a patient to the hospital?
7. Are you familiar with the “diagnostic loop”? If yes, can you please tell me how you use this service?

Medical quality

1. What do you think about the quality of the medical treatment patients get at a MAW?
2. What do you think about the quality of the medical treatment patients receive at a hospital?

Follow up: What is included in good quality? What is something else that leads to quality?

1. When in doubt, which factors make you decide to admit the patient?
2. Which factors make you feel safe not to admit the patient?

User involvement

Could you please describe a situation when the patient did not want to be admitted, even if you wanted it? Follow up: How did you act in this situation?

To what extent do you include patients’ desires about treatment in your decision?

To what extent do you feel that the system lets you include patients’ desires?

The MAW model in healthcare services

1. What do you think about the MAW as a future healthcare service?

Is there something else relating to MAW services that you would like to add?
